# Association between Clinical/Radiographic Characteristics and Histopathological Diagnoses of Periapical Granuloma and Cyst

**DOI:** 10.1055/s-0042-1759489

**Published:** 2023-01-04

**Authors:** Danuchit Banomyong, Raweewan Arayasantiparb, Kanoknuch Sirakulwat, Jane Kasemsuwan, Nannaphat Chirarom, Nithiporn Laopan, Puangwan Lapthanasupkul

**Affiliations:** 1 Department of Operative Dentistry and Endodontics, Faculty of Dentistry, Mahidol University, Bangkok, Thailand; 2 Dental Department, Chulabhorn Hospital, Chulabhorn Royal Academy, Bangkok, Thailand; 3 Department of Oral and Maxillofacial Radiology, Faculty of Dentistry, Mahidol University, Bangkok, Thailand; 4 Faculty of Dentistry, Mahidol University, Bangkok, Thailand; 5 Department of Oral and Maxillofacial Pathology, Faculty of Dentistry, Mahidol University, Bangkok, Thailand

**Keywords:** dental radiograph, endodontic microsurgery, granuloma, histopathology, periapical cyst

## Abstract

**Objective** The aim of this study was to determine the association between clinical/radiographic characteristics and histopathological diagnoses of periapical granuloma and cyst obtained from the teeth treated with endodontic microsurgery.

**Materials and Methods** The clinical, radiographic (periapical and cone-beam computed tomography), and histopathological data were collected from patients' dental records of endodontic microsurgery on the teeth with periapical lesions. These lesions were histopathologically diagnosed as either granuloma or cyst, at the Endodontic Clinic, Faculty of Dentistry, Mahidol University, Bangkok, Thailand, from 2016 to 2021 according to inclusion and exclusion criteria. The data were analyzed using bivariate analysis and a multinomial logistic regression at a significance level of *p* -value less than 0.05.

**Results** A total of 83 patients (58 females and 25 males) with an average age of 45 to 49.5 years old met the criteria including 68 periapical granulomas (81.9%) and 15 periapical cysts (18.1%). A periapical lesion involving multiple teeth in a periapical radiograph was significantly associated with the histopathological diagnosis of periapical cyst ( *p* < 0.05). Such periapical lesion was six times more likely to be periapical cyst than periapical granuloma.

**Conclusions** A significant factor for predicting periapical cyst from periapical granuloma was the presence of a periapical lesion with multiple-teeth involvement in a dental radiograph.

## Introduction

A periapical lesion in a necrotic tooth is induced by microbial infection from the root canal system, which results in an inflammatory reaction and alveolar bone destruction. A periapical diagnosis can be symptomatic/asymptomatic apical periodontitis or chronic/acute apical abscess depending on clinical and radiographic characteristics. The histopathological diagnosis of periapical lesion includes granuloma (nonepithelialized or epithelialized), cyst, abscess, or others (e.g., scar or foreign body reaction) depending on the microstructure in the biopsy specimens. 1 2 With regard to the previous histopathologic studies, the prevalence of periapical granulomas (PGs) and periapical cysts (PCs) were 48 to 85 and 23.5 to 42%, respectively, while the periapical abscess was reported in a few studies with a wide range of prevalences. 1 2 3 4 5 6 7

From the histopathological point of view, the granulation tissue infiltrated with chronic inflammatory cells is initially formed in the periapical area of bone destruction. 8 When the periapical lesion is growing, the growth may stimulate the proliferation of epithelial rests to encircle the lesion. The center of granuloma begins to lack blood supply and is gradually degenerated. A cystic cavity is then formed as a periapical cyst with epithelial lining and fluid content inside. 8 The difference in osmolarity induces tissue fluid diffusion into the cystic cavity, and the inflammatory mediators involving bone resorption increase an expansion of the cystic lesion. Hence, the PC tends to be larger than the PG; however, the size of granuloma may be also large. 9 10 For a periapical abscess, the lesion contains the granulation tissue with acute inflammatory cells such as polymorphonuclear leukocytes (neutrophils) or plasma cells, which is possibly an early infection stage of the PG. 1 11 When the acute stage is subsided, the periapical abscess may turn to the PC if the proliferation of epithelial lining is formed. 12

The necrotized tooth with periapical lesion is primarily treated by nonsurgical root canal treatment. In some circumstances, a failure of root canal treatment can be developed due to several factors such as persistence of bacteria or inadequate filling of the canal. 13 An advanced treatment of endodontic microsurgery is then required. The PG is more likely to be healed after the nonsurgical treatment compared to the PC. 12 Nevertheless, many large cysts can be healed by apoptosis after root canal treatment. 9 14 A “pocket” cyst that shows a communication to the apical foramen frequently responds to the treatment after the source of infection from the root canal is eliminated. 12 In contrast, a “true” cyst with complete epithelial lining and no communication to the apical foramen is possibly not healed after the conservative treatment, in which surgical removal by endodontic microsurgery may be later required. 12 However, the PG and the pocket/true cyst can be only diagnosed from the histopathological examination of the biopsy specimens.

Many studies attempted to find an association between clinical/radiographic data and histopathological diagnoses of PG and PC. 6 10 15 16 17 18 19 20 From the clinical presentation, the PC may be associated with detection of fluid content draining through the root canal or sinus tract. 21 22 On the other hand, characteristics of periapical radiolucency in dental radiographs and/or cone-beam computed tomography (CBCT) may be used to predict the PCs, for example, a presence of a radiopaque rim (corticated border), size (such as ≥10 mm or 200 mm ^2^ ) of lesions, or volume (such as ≥ 250 mm ^3^ )/characteristics of lesions in CBCT. 6 20 23 However, the prediction of histopathology of periapical lesions based on these clinical and/or radiographic appearances is controversial. 1 3 10 16 17 18 19

Therefore, this retrospective study aimed to determine the association between histopathological diagnoses and clinical/radiographic appearances of PGs and PCs obtained from the teeth treated with endodontic microsurgery.

## Materials and Methods

The protocol of the study was approved by the institutional ethic committee (MU-DT/PY-IRB 2021/075.2308). The clinical, radiographic, and histological information were collected from the teeth treated with endodontic microsurgery at the Faculty of Dentistry, Mahidol University, Bangkok, Thailand from 2016 to 2021. All teeth were treated by endodontists using microsurgical technique. The soft tissue of periapical lesions were removed during the surgery and delivered to pathologists for interpretation. The inclusion criterion was the teeth with histopathological reports of PG or PC. The exclusion criteria were (a) inadequate clinical record, (b) absence of radiographic data, (c) unclear histopathological diagnosis, and (d) other histopathologic diagnoses, that is, periapical abscess, scar, or foreign body reaction.

### Data Acquisition

The clinical information were collected as follows: sex (male/female); age (years old); site of lesion (anterior maxilla/posterior maxilla/anterior mandible/posterior mandible); type of teeth (incisor /canine/ premolar/molar); history or presence of these parameters (yes/no)—sinus opening, swelling, pus, or exudate; tooth mobility (yes/no); pain on percussion (yes/no); and clinical diagnosis (symptomatic/asymptomatic apical periodontitis, acute/chronic apical abscess).

The digital periapical (parallel) radiographs (X-Mind, Acteon, Olgiate Olona (VA), Italy) were taken before the surgery. CBCT (3D Accuitomo 170, J. Morita, Kyoto, Japan) was occasionally taken in a few cases depending on the operators' decision. The radiographic data of periapical lesions were collected including lesion size (<1 cm, ≥1 cm); radiopaque rim (fully, partially, without); margin (well-defined, ill-defined); root resorption (yes, no); and multiple-teeth involvement (yes, no). In addition, the data of periapical lesions were collected from CBCT (if presented) including the perforation of cortical bone (with, without), and volume of the lesion (mm ^3^ ). The radiographic evaluation of periapical lesions was performed by a qualified oral and maxillofacial radiologist (RA). The evaluation of intraobserver reliability revealed intraclass correlation coefficient ranging from 82 to 98% that indicated good reliability.

The histopathological diagnoses were obtained from the specimens prepared by the serial-section technique. The specimens were microscopically re-examined and classified as PG or PC by a certified oral pathologist (PL) according to the criteria modified from Nobuhara and Del Rio 24 and Ricucci et al 25 ; the presence of a cystic cavity or granulation tissues, detection of epithelium lining, and any content in the cavity. The PG contained granulation tissue with no epithelial lining (or with minor epithelial strand) and no cavity or fluid content. The PC had a cystic cavity with major epithelium lining and occasionally contained cellular debris within the cavity.

### Statistical Analysis

The statistical analysis was performed using STATA version 17 (StataCorp LLC, College Station, Texas, United States). The descriptive statistics were used to describe the distribution of periapical granulomas and cysts according to the considerable factors. For the radiographic evaluation, the intraobserver reliability was assessed by intraclass correlation coefficient. The association between clinical /radiographic appearances and histopathological diagnoses was determined using the bivariate analysis (chi-squared and Fisher's exact test) and followed by the multinomial logistic regression analysis.

## Results

**Fig. 1:**
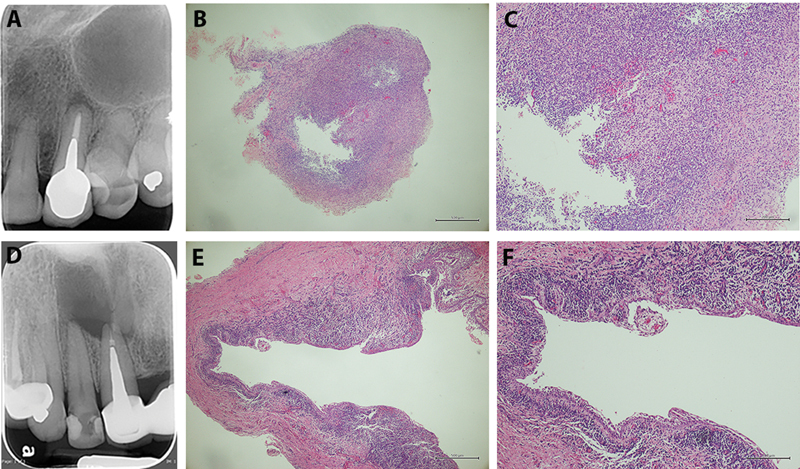
The *upper* row—the representative radiograph ( **A** ) and the histopathology ( **B** , **C** at 40x and 200x magnifications) of periapical granuloma. The *lower* row—the representative radiograph ( **D** ) and the histopathology ( **E** , **F** at 40x and 200x magnifications) of periapical cyst.

A total of 111 teeth were initially included according to the inclusion criteria, and then 28 teeth were excluded according to the exclusion criteria. A total of 83 teeth remained for data analysis, of which 68 periapical lesions (18.1%) were granulomas and 15 periapical lesions (81.9%) were PCs. The 83 teeth were obtained from 83 patients: 25 males and 58 females aged between 14 and 75 years old with a mean age of 48.7 ± 13.8 years. The majority of the teeth were the incisors in the anterior maxillary region. All general information, clinical, radiographic, and cone-beam CT data are shown according to the PG and cyst groups in Tables 1 2 3 . Representatives of the radiographs and histopathology of the PG and PC are presented in Fig.1 .

**Table 1:** The general information of the periapical granuloma group and the periapical cyst group (total, *n* = 83)

General information	Periapical granuloma ( *n* = 68)	Periapical cyst ( *n* = 15)
**Age** (mean ± SD, years)	49.5 ± 13.7	45.0 ± 14.0
**Gender**		
Male	20 (29.4%)	5 (33.3%)
Female	48 (70.6%)	10 (66.7%)
**Tooth type**		
Incisor ( *n* = 65)	52 (76.5%)	13 (86.7%)
Canine ( *n* = 3)	3 (4.4%)	0
First premolar ( *n* = 9)	9 (13.2%)	0
Molar( *n* = 5)	3 (4.4%)	2 (13.3%)
Combination ( *n* = 1)	1 (1.5%)	0
** Site of lesion**		
Anterior maxilla ( *n* = 63)	51 (75%)	12 (80%)
Posterior maxilla ( *n* = 13)	11 (16.2%)	2 (13.3%)
Anterior mandible ( *n* = 6)	5 (7.3%)	1 (6.7%)
Posterior mandible ( *n* = 1)	1 (1.5%)	0

Abbreviation: SD, standard deviation.

**Table 2:** The clinical and radiographic information of the periapical granuloma group and the periapical cyst group (total, *n* = 83) with a bivariate analysis (chi-squared and Fisher's exact test)

Factors	Periapical granuloma ( *n* = 68)	Periapical cyst ( *n* = 15)	Bivariate analysis ( *p* -Value)
**Clinical appearances**			
**History of fistula**			
Yes ( *n* = 36)	32 (47.1%)	4 (26.7%)	0.149 a
No ( *n* = 47)	36 (52.9%)	11 (73.3%)	
**History of swelling**			0.108 a
Yes ( *n* = 23)	16 (23.5%)	7 (46.7%)	
No ( *n* = 60)	52 (76.5%)	8 (53.3%)	
**History of pus**			1
Yes ( *n* = 17)	14 (20.6%)	3 (20%)	
No ( *n* = 66)	54 (79.4%)	12 (80%)	
**History of exudate**			0.003 a
Yes ( *n* = 5)	1 (1.5%)	4 (26.7%)	
No (n = 78)	67 (98.5%)	11 (73.3%)	
**Tooth mobility** ( *n* = 77) b			0.617
Yes ( *n* = 7)	5 (8.1%)	2 (13.3%)	
No ( *n* = 70)	57 (91.9%)	13 (86.7%)	
**Pain on percussion** ( *n* = 81) b			0.358
Yes ( *n* = 24)	18 (27.3%)	6 (40%)	
No ( *n* = 57)	48 (72.7%)	9 (60%)	
**Clinical diagnosis**			
AAP ( *n* = 22)	17 (25%)	5 (33.3%)	0.633
SAP ( *n* = 9)	8 (11.8%)	1 (6.7%)	
AAA ( *n* = 15)	11 (16.2%)	4 (26.7%)	
CAA ( *n* = 37)	32 (47%)	5 (33.3%)	
**Radiographic appearances**			
**Tooth involvement**			0.002 a
Single tooth ( *n* = 73)	64 (94.1%)	9 (60%)	
Multiple teeth ( *n* = 10)	4 (5.9%)	6 (40%)	
** Size of lesion**			0.003 a
< 1 cm ( *n* =50)	46 (67.6%)	4 (26.7%)	
≥ 1 cm ( *n* = 33)	22 (32.4%)	11 (73.3%)	
**Margin**			0.212 a
Well-defined ( *n* = 58)	45 (66.2%)	13 (86.7%)	
Ill-defined ( *n* = 25)	23 (33.8%)	2 (13.3%)	
**Radiopaque rim**			0.149 a
None ( *n* = 57)	49 (72.1%)	8 (53.3%)	
Partial ( *n* = 8)	7 (10.3%)	1 (6.7%)	
Full ( *n* = 18)	12 (17.6%)	6 (40%)	
** Root resorption** ( *n* = 79) b			0.591
Yes ( *n* = 40)	32 (49.2%)	8 (57.1%)	
No ( *n* = 39)	33 (50.8%)	6 (42.9%)	

Abbreviations: AAA, acute apical abscess; AAP, asymptomatic apical periodontitis; CAA, chronic apical abscess; SAP, symptomatic apical periodontitis.

a The factors with a *p* -value≤0.25 were further included in the multinomial logistic regression model.

b Data was not available in all cases.

**Table 3:** The cone-beam computed tomography information of the periapical granuloma group and the periapical cyst group (total, *n* = 23)

Cone-beam computed tomography factors	Periapical granuloma ( *n* = 21)	Periapical cyst ( *n* = 2)
**Volume of lesions**		
Mean	222.1 ± 270.7 mm ^3^	2238.5 ± 789.1 mm ^3^
Range	6.678-882.518 mm ^3^	1680.5- 2796.404 mm ^3^
Perforation of cortical bone ( *n* )	15 (71.4%)	2 (100%)

For the clinical appearances ( Table 2 ), the PC group showed a higher prevalence of history of swelling or exudate compared to the PG group—46.7 versus 23.5% and 26.7 versus 1.5%, respectively. In contrast, the PC group showed a lower prevalence of history of fistula (26.7%) compared to the PG group (47.1%). However, the prevalence of history of pus was the same between the two groups (20% approximately). The common clinical diagnoses in both groups were chronic apical abscess and asymptomatic apical periodontitis.

For the radiographic appearances ( Table 2 ), the PC and PG groups typically showed different characteristics. The PC group had a higher prevalence than the PG group in multiple-teeth involvement (40 vs. 5.9%), a well-defined margin (86.7 vs. 66.2%), and partial/full radiopaque rim (46.7 vs. 27.9%). Moreover, the lesion size of the PC group was commonly larger than 1 cm (73.3%), while the lesion size of the PG group was frequently smaller than 1 cm (67.6%). Approximately half of either PC or PG group had a sign of root resorption.

The CBCT data was available only in 23 out of 83 teeth ( Table 3 ), including 2 teeth in the PC group and 21 teeth in the PG group. The mean volume of PC was 2238.5 ± 789.1 mm ^3^ while that of PG was 222.1 ± 270.7 mm ^3^ . Moreover, 100% of PC presented the perforation of cortical bone, whereas 71.4% of PG did.

The results of the bivariate analysis are presented in Table 2 . In comparison to the PG group, the factors probably associated with the diagnosis of PC are the three clinical appearances including history of fistula, swelling, and exudate, as well as the four radiographic appearances including multiple-teeth involvement, lesion size more than 1 cm, well-defined margin, and radiopaque rim ( *p* < 0.25). However, the history of exudate was excluded from the multinomial logistic regression model since only one case was observed in the PG group that was not appropriate for further statistical analysis. Among the remaining factors, the multiple-teeth involvement was only a significant factor in the multinomial logistic regression analysis ( *p* < 0.05; Table 4 ). The periapical lesion with the radiographic appearance of multiple-teeth involvement showed approximately six times higher chance to be PC than PG.

**Table 4:** The multinomial logistic regression analysis of the factors selected from the bivariate analysis as a predictor of periapical cyst compared to periapical granuloma

Factors	Relative risk ratio	*p* -Value	95% confidence interval
History of swelling	1.560	0.661	0.212 − 11.445
History of fistula	1.334	0.728	0.262 − 6.789
Margin (well-defined)	1.063	0.951	0.150 − 7.510
Radiopaque rim (yes)	1.432	0.414	0.604 − 3.393
Size of lesion (≥1 cm)	3.015	0.175	0.612 − 14.843
Tooth involvement (multiple)	6.307	0.035 a	1.139 − 34.903

a Multiple-teeth involvement was only the significant factor with a *p* -value < 0.05 and a relative risk ratio of 6.307.

## Discussion

This retrospective study is one of a few clinical studies comprehensively evaluating an association between clinical/radiographic data and histopathological diagnoses of periapical lesions. Most of the other studies only investigated the association from either clinical or radiographic data. 1 16 17 18 26 27

The multiple-teeth involvement in periapical radiographs was significantly associated with the diagnosis of PC. This can be explained by the pathogenesis of PG and PC, in which PC tends to be larger than PG. 21 22 The prolonged inflammation of PG may induce the proliferation and differentiation of the epithelial rests of Malassez to form the epithelial lining that encloses the highly vascularized granulation tissue. After that, the center of granulation tissue lacks blood supply that consequently induces liquefaction necrosis and becomes a cystic cavity with enclosing epithelial lining. The osmolarity of cystic fluid contributes to the increase of fluid content via transportation of serum fluid from the neighboring tissue accumulating into the cystic cavity, and, as a result, the enlargement of PC especially in the horizontal dimension. 14 In our study, the prediction of PC was significantly related to the multiple-teeth involvement with sixfolds higher chance than that of PG.

The radiographic size of periapical lesion at or greater than 1 cm was not a significant predictor for the cyst in our study. Other studies 10 17 19 also failed to show an association between the histopathological diagnosis of the PC and the lesion size. In contrast, Mortensen et al 15 and Natkin et al 6 proposed that PC could be differentiated from PG when the size of the lesion was equal to or larger than 1 cm. This discrepancy maybe due to a difference of the sample size and different study cohorts. However, the prediction of PC from the radiographic size of the lesion remains controversial and may be unreliable. 10 19 28 However, the horizontal enlargement of periapical lesion involving the adjacent tooth was a significant predictor in our study. This result may be explained by the tendency of cystic expansion in the horizontal dimension rather than in the vertical dimension, as previously mentioned.

It was believed that a radiopaque rim around the periapical lesion was the bone reaction, as a defense mechanism, to enclose the slow-progressing cystic lesion. However, the present study did not find a significant association between the radiopaque rim and the histological diagnosis of PC, corresponding to the results of Ricucci et al. 16

Most of the clinical studies have reported no relationship between clinical characteristics and histopathological diagnoses of PC or granuloma. 3 7 26 28 In our study, the history of inflammatory exudate tended to be a predictor of the PC; however, the number of cases with the exudate in our study was limited (only 5 teeth; 1/68 teeth in the granuloma group, and 4/15 teeth in the cyst group) and not appropriate for statistical analysis. The exudate is commonly the content in the cystic cavity and may be used as a predictor for the PC. 22 Our retrospective study collected the data from dental records, in which the presence of minor exudate may be neglected and not documented.

Our study found a trend in the three-dimensional CBCT images that the PC (2238 ± 789 mm ^3^ ) had a higher volume than the PG (222 ± 270.7 mm ^3^ ), which was close to what was reported in a previous study. 20 We believed that there is a tendency to use the CBCT volume of a periapical lesion for predicting the PC. However, the number of cases with CBCT data in our study was limited due to preoperative CBCT that has not been yet a standard of examination before endodontic microsurgery in our daily practice. A relationship between the CBCT volume and the histopathology of the periapical lesion should be further investigated.

Based on our findings, the multiple-teeth involvement may be used as a predictor to distinguish between the PG and PC, that probably imply the prognosis of treatment. The PG is commonly healed after nonsurgical root canal treatment, and the PC is also likely to be healed after the nonsurgical treatment by the apoptosis of epithelial lining. 14 However, it has been proposed that the pocket cyst (with a communication to the root canal) can be healed by the root canal treatment, while a true cyst (completely isolated from the root canal) is self-sustaining and less likely to respond to the nonsurgical treatment. 12 A possible association (if any) between the clinical/radiographic findings and the histological diagnosis may be useful to predict the prognosis of root canal treatment.

## Conclusion

In our studied population, the incidences of PG and cyst were approximately 82 and 18%, respectively. The significant factor for prediction of PC was the multiple-teeth involvement of the periapical lesion in a dental radiograph.
